# Impact of the revision of a nutrient database on the validity of a self-administered food frequency questionnaire (FFQ)

**DOI:** 10.2188/jea.16.107

**Published:** 2006-05-19

**Authors:** Junko Ishihara, Manami Inoue, Minatsu Kobayashi, Sachiko Tanaka, Seiichiro Yamamoto, Hiroyasu Iso, Shoichiro Tsugane

**Affiliations:** 1 Epidemiology and Prevention Division, Research Center for Cancer Prevention and Screening, National Cancer Center.; 2 Department of Public Health Medicine, Doctoral Program in Social and Environmental Medicine, Graduate School of Comprehensive Human Sciences, University of Tsukuba.; 3 Department of Domestic Science, Otsuma Women’s University.; 4 Statistics and Cancer Control Division, Research Center for Cancer Prevention and Screening, National Cancer Center.; 5 Public Health, Department of Social and Environmental Medicine, Graduate School of Medicine, Osaka University.

**Keywords:** Eating, Questionnaires, food composition table, validity, JPHC Study

## Abstract

**BACKGROUND:**

Revision of the national nutrient database in 2000 had a strong impact on the absolute level of estimated nutrient intake in dietary assessments. However, whether it influenced the ranking of individuals by estimated intake, a more important function in epidemiologic studies, has not been investigated. Here, we investigated the effect of this revision of the nutrient database on the validity of a food frequency questionnaire (FFQ) used to estimate nutrient intake in the Japan Public Health Center-based prospective Study (JPHC Study).

**METHODS:**

Subjects were a subsample of the JPHC Study who volunteered to participate in the validation study of the FFQ. Validity of the FFQ was evaluated by reference to the 28-day weighed dietary records as a gold standard. Nutrient intake according to the FFQ was recalculated using the revised database, and the results were compared to those using the previous database. Spearman’s rank correlation coefficients (CCs) between intakes estimated by the FFQ and dietary records were computed using the revised database, and were compared to CCs computed using the previous database.

**RESULTS:**

For most of the nutrients, mean intake increased or decreased significantly using the revised database. However, no notable change was seen for the CC between estimated intake according to dietary records and FFQ when the revised database was used for calculation. Differences in the point estimates of the CCs ranged from -0.14 to 0.15. Likewise, CCs between biomarkers and estimated intake according to FFQ were similar for the two databases.

**CONCLUSION:**

Despite changes in intake levels for many nutrients, the validity of our FFQ using rank correlation by nutrient intake was not influenced by revision of the nutrient database in Japan.

In epidemiologic studies, dietary intake is often assessed by means of food frequency questionnaires (FFQs), thanks to their ease of administration and low burden on the subject.^1^ In epidemiologic applications, individuals are often classified into groups by estimated intake, and such classification is most often the primary objective of an FFQ.^1^ The ability of an FFQ to rank individuals by estimated intake is therefore important.

When calculating the individual nutrient intake from foods estimated by an FFQ, food composition databases are used as a source of nutrient contents. Given the variation among databases, database selection would affect the results of individual nutrient intakes greatly. The Standard Tables of Food Composition in Japan, published by the Ministry of Education, Culture, Sports, Science and Technology, is the most commonly used food composition database in Japan. It lists the nutrient contents of various foods per 100g which are average and representative values among those foods available in Japan. The database has been revised on an irregular basis. The Fifth Revised Edition was released in 2000, almost 20 years after the Revised Fourth Edition,^2^ and an Enlarged Edition covering additional nutrients was released in 2005.^3^ The database was revised to update the nutrient content of a greater variety of food items commonly eaten by Japanese, which have changed over time with changes in manufacture and distribution in the food industry.^3^ Further, the revised database is more comprehensive, including additional nutrients not listed in the previous database. This is greatly beneficial when associations with disease are investigated because it allows the estimation of exposure to specific nutrients of interest.

This revision of the nutrient database, however, has greatly influenced the estimation of intakes in the National Nutrition Survey (NNS) in Japan.^4^ A decline assumed to be attributable to the revision was observed in average intake for a number of nutrients including iron, vitamin B_1_, vitamin B_2_, and vitamin C. Other studies have reported that the degree of difference between the previous and current editions varies by age group.^5^^,^^6^ Nevertheless, it remains unknown whether the revision of the food composition tables has had an effect on the validity of any of the various FFQs, and the validity of the intake of nutrients newly added in the Enlarged Edition of the Fifth Revised Edition has never been evaluated. Indeed, we are unaware of any previous study which has evaluated the impact of a revision of a nutrient database on the validity of an FFQ.

Here, to investigate the effect of the revision of the food composition tables on the validity of an FFQ, we compared the ranking of individuals by estimated nutrient intake calculated using the revised database (Fifth Edition) to that using the previous database (Fourth Edition) in a subgroup of the Japan Public Health Center-based Prospective Study (JPHC Study) using dietary records (DRs) and biomarkers as references. Additionally, we also evaluated the validity of the FFQ in estimating the intake of nutrients newly included in the Enlarged Edition of the Fifth Revised Edition.

## METHODS

### Study Setting

The JPHC Study is a population-based prospective cohort study which consists of two cohorts, the first established in 1990 in the Ninohe, Yokote, Saku, and Chubu (previously named Ishikawa) public health center areas (Cohort I), and the second in 1993 in the Mito, Kashiwazaki, Chuo-higashi, Kamigoto, Miyako and Suita public health center areas (Cohort II). The aim of the cohort study was to investigate associations between chronic diseases and various lifestyle factors such as diet. The study design and participants in the overall cohort have been described previously.^7^ To assess the dietary intake of individuals in these populations, a semi-quantitative FFQ was developed based on data from 3-day weighed DRs in a random sample from Cohort I.^8^

Two FFQ validation studies were conducted in subsamples of Cohort I and Cohort II, started February 1994 and May 1996, respectively. The purpose of the study in Cohort I subjects was to validate the FFQ within the population for which the FFQ was developed, while that in Cohort II was to evaluate the validity of the FFQ in a population which was not that for which the FFQ was developed (external validity). Approximately 30 married couples age 45 to 74 each were recruited through the respective public health centers.^9^^,^^10^ Mean ages of Cohort I subjects were 55.6 and 54.6 years for males and females, respectively, while those of Cohort II were 58.9 and 55.9 years, respectively. Subjects from both Cohorts were healthy volunteers without dietary restrictions and they were not over- or underweight. Company-employed workers and housewives were the most common occupation among males and females, respectively.

### Data Collection

**Figure 1.**
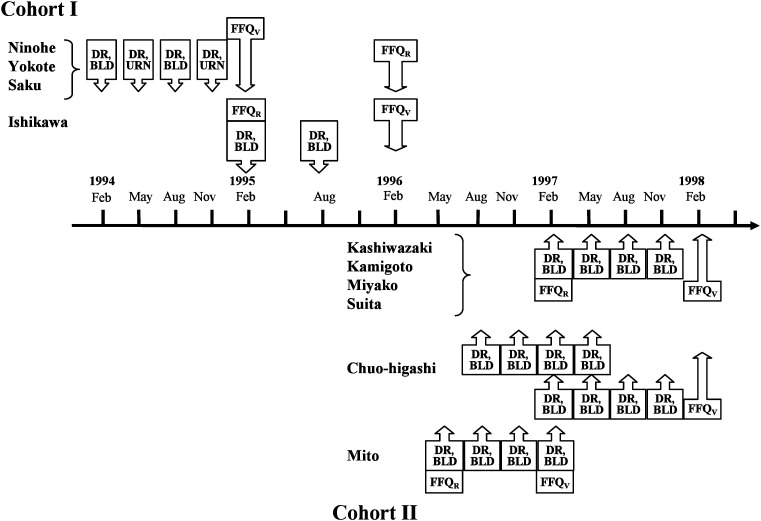
Data collection sequence in the JPHC FFQ Validation Study. DR: 28-day dietary records FFQ_V_: food frequency questionnaire for validity FFQ_R_: food frequency questionnaire for reproducibility BLD: blood collection; URN: urine collection

Data collection has been described in detail elsewhere.^9^^,^^10^ In brief, each subject completed 28-day DRs and two identical FFQs (FFQ_V_ and FFQ_R_), conducted for different purposes (Figure 1): the FFQ_V_ was completed immediately or 3 months after the 28-day DRs were obtained to provide the data required for comparison with the DRs, while the FFQ_R_ was administered to provide data to evaluate the reproducibility of FFQ_V_. For validity, we analyzed the data of 215 and 350 subjects in Cohorts I and II, respectively, who had complete data for the 28-day DRs and the second FFQ (FFQ_V_). For reproducibility, we analyzed the data of 209 and 289 subjects in Cohorts I and II, respectively, who had complete data for the both FFQs. Fasting blood, 24-hour stored urine or both were also collected from Cohort I and II subjects, with some of these samples from Cohort I subjects analyzed for serum phospholipids (saturated, monounsaturated and polyunsaturated fatty acids) and carotenoids (alpha-carotene, beta-carotene, cryptoxanthin), plasma vitamin B_6_, vitamin B_12_, folate, and vitamin C, and urinary sodium and potassium, and the results were compared with intake levels.

The DRs were collected over 7 consecutive days in each of the 4 seasons, except in Chubu (2 seasons). Local dietitians instructed the subjects to weigh all foods and beverages with the scales and measuring utensils provided, and to record the results in a specially designed booklet. The subjects in Cohort I, however, were instructed to use standardized portion sizes for some foods that were difficult to weigh (semi-weighed DRs). The subjects described each food, method of preparation, and the name of the dish in detail. They also reported all dietary supplements used, if any. At the end of each season, the DRs were reviewed in a standardized manner, and each food was coded using the food item code in the Standardized Tables of Food Composition, 4th ed.^2^ by local dietitians. Energy and nutrient intake were calculated by summing the product of the intake of each food multiplied by the nutrient content of that food. The nutrients listed in the Standardized Tables of Food Composition, 4th ed. were protein, total fat, carbohydrate, sodium, potassium, calcium, phosphorus, iron, retinol, vitamin B_1_, vitamin B_2_, niacin, and vitamin C. Additionally, for those nutrients with missing values for some foods, i.e., carotenes (alpha- and beta-),^11^ fatty acids (saturated, monounsaturated and saturated),^12^ cholesterol, and dietary fiber (soluble, insoluble and total),^13^ a comprehensive database was developed by substitution methods.

The self-administered semi-quantitative FFQ consisted of 138 food items and 14 supplementary questions concerning the use of dietary supplements, dietary habits, and others. Results were used to assess the usual dietary intake of the preceding year for each individual. The intake of each food item was calculated by multiplying the frequency of consumption (never, 1-3 times/months, 1-2 times/week, 3-4 times/week, 5-6 times/week, once/day, 1-2 times/day, 4-6 times/day, 7+ times/day) by relative portion size (small, medium, and large). The food item code in the Standardized Tables of Food Composition, 4th ed.^2^ was also assigned for each food item in the FFQ,^14^ and daily intake of energy and nutrients according to the FFQs for each individual were calculated by summing the product of the intake of each food multiplied by the nutrient content of that food for the same nutrients which were calculated for dietary records. In addition, folate, vitamin B_6_, and vitamin B_12_ intake were calculated using the database developed for the food items which appeared on the FFQ.^15^ Because a database of dietary supplements was not available, intake from dietary supplements was not included in calculations for both DR and FFQ.

Energy and nutrient intake according to the FFQ and DR were then recalculated using the Standardized Tables of Food Composition, 5th ed. (revised database).^3^ The 4th edition (previous database), which was published in 1982, included values for energy, protein, fat, carbohydrate, sodium, potassium, calcium, phosphorus, iron, retinol, carotene, vitamin B_1_, vitamin B_2_, niacin, and vitamin C of 1621 food items. Continuously thereafter, values for amino acids, fatty acids, cholesterol, vitamin E, magnesium, zinc, copper, dietary fiber, and vitamins D, K, B_6_, and B_12_ were published, but only for some major food items, rather than all 1621 food items. The various databases were integrated in the revised database, published in 2000, which also included a greater variety of food items (1,878 foods). This database provided food composition values for some nutrients which were not presented in the previous database, such as retinol equivalents, beta-carotene equivalents, cryptoxanthin, pantothenic acid, and NaCl deducted from sodium content. It also provided food composition values for all 1,878 food items for those nutrients for which values were only available for some foods in the previous database, such as magnesium, zinc, copper, vitamins D, E, K, B_6_, and B_12_, and folate. For all food item codes in the previous database that appeared in the DR and FFQs, equivalent food item codes in the revised database were assigned. When an exactly equivalent food item was not available, an alternative item of close botanical or zoological relevance was taken as a surrogate.

### Statistical Analysis

The mean intakes of energy and nutrients according to the FFQs were calculated by sex for Cohorts I and II using the previous and revised databases. Intake levels based on the revised database were compared with those based on the previous database by means of mean difference (in which intake calculated with the previous database is subtracted from that with revised database), and percentage of changes (in which mean difference is divided by intake calculated using the previous database). Statistical differences between intake levels based on the two databases were tested by Student’s paired t-tests.

Validity of the FFQ in the estimation of crude and energy-adjusted intake (residual method) was evaluated by Spearman’s rank correlation coefficients (CCs) using mean intake from the 28-day DR and biomarkers as references. In addition, reproducibility of the FFQ for the estimation of crude and energy-adjusted intake (residual method) was evaluated by the Spearman’s rank CCs between intake levels according to the two FFQs administered at different times. These CCs were compared to the respective CCs calculated using the previous database using the point estimate and its 95% confidence interval of each CC. All analyses were performed using SAS^®^ Version 9.1 (SAS Institute Inc., Cary, NC).

## RESULTS

Mean nutrient intakes by the FFQ calculated using the previous and revised databases are shown in Table 1. Differences in estimated intake as a result of the revision were not particularly apparent for macronutrients, but were more apparent for micronutrients. Intakes of all minerals were estimated to be lower with the revised database, most evidently for iron (-8.3% to -12.5%). The impact of the database revision was more obvious for vitamins; among these, intake of carotenes and retinol was 55% and 12.5% higher, respectively, whereas that of B group vitamins was lower. For nutrients for which we supplemented missing values in the database, intake of monounsaturated fatty acid was lower after the database revision, while that of water-soluble fiber was drastically higher.

**Table 1.** Daily nutrient intake assessed with a self-administered food frequency questionnaire calculated using the previous and revised databases.

	Cohort I										Cohort II								
	
	Previous database*			Revised database^†^			Mean difference^‡^	% changes^§^	P-value^∥^		Previous database*			Revised database^†^			Mean difference^‡^	% changes^§^	P-value^∥^
					
	Mean	SD	Median	Mean	SD	Median					Mean	SD	Median	Mean	SD	Median			
										Male									
	n=102										n=174								
Energy (kcal)	2313	693	2274	2313	665	2263	1	0.4	0.92		2148	636	2091	2196	648	2126	48	2.3	<0.001
Protein (g)	86.7	35.9	78.0	81.4	32.2	75.4	-5.3	-5.6	<0.001		77.0	29.3	72.9	75.6	28.7	71.2	-1.5	-1.9	<0.001
Total fat (g)	63.6	27.7	59.9	61.4	26.4	58.0	-2.2	-3.1	<0.001		59.5	25.0	54.7	59.8	25.7	54.8	0.4	0.3	0.02
Carbohydrate (g)	304	100	291	306	100	296	3	1.0	0.01		281	83	267	285	85	271	5	1.6	<0.001
Sodium (mg)	5615	2608	5356	5309	2384	5302	-306	-4.2	<0.001		4941	2529	4344	4577	2162	3990	-365	-6.1	<0.001
Potassium (mg)	3212	1483	3001	3138	1430	2910	-74	-2.2	<0.001		2996	1332	2691	2929	1279	2602	-67	-2.0	<0.001
Calcium (mg)	656	393	591	600	362	554	-57	-7.7	<0.001		612	365	529	593	370	506	-19	-3.9	<0.001
Phosphorus (mg)	1380	555	1329	1269	502	1234	-110	-7.7	<0.001		1250	468	1156	1195	467	1088	-55	-4.8	<0.001
Iron (mg)	11.8	5.2	11.1	10.5	4.0	10.2	-1.3	-8.9	<0.001		10.2	4.0	9.5	9.2	3.3	8.4	-0.9	-8.3	<0.001
Retinol (*μ*g)	619	566	503	644	566	521	25	7.1	<0.001		515	390	459	544	397	482	29	8.9	<0.001
Alpha-carotene (*μ*g)	561	545	398	732	745	539	171	31.3	<0.001		520	544	403	708	758	539	188	36.6	<0.001
Beta-carotene (*μ*g)	3044	2582	2556	4445	3613	3815	1401	48.6	<0.001		2715	2305	2202	4088	3346	3331	1373	53.6	<0.001
Vitamin B_1_ (mg)	1.24	0.50	1.11	1.12	0.51	0.99	-0.11	-10.0	<0.001		1.24	0.58	1.12	1.11	0.55	0.99	-0.13	-11.4	<0.001
Vitamin B_2_ (mg)	1.72	0.75	1.62	1.64	0.72	1.53	-0.08	-4.9	<0.001		1.62	0.73	1.44	1.52	0.70	1.33	-0.11	-6.5	<0.001
Niacin (mg)	20.5	8.3	18.5	22.7	9.1	20.6	2.2	10.9	<0.001		18.6	7.9	17.4	20.5	8.7	18.6	1.8	9.9	<0.001
Vitamin B_6_ (mg)	1.98	0.80	1.81	1.81	0.75	1.66	-0.17	-8.8	<0.001		1.82	0.75	1.71	1.65	0.68	1.55	-0.17	-9.2	<0.001
Vitamin B_12_ (*μ*g)	12.2	8.5	9.8	11.0	7.5	8.8	-1.2	-7.4	<0.001		10.6	6.3	8.8	9.5	5.5	8.1	-1.1	-9.3	<0.001
Folate (*μ*g)	319	152	288	473	231	444	154	48.0	<0.001		297	134	272	421	190	370	124	42.1	<0.001
Vitamin C (mg)	166	118	157	159	108	148	-7	-3.5	<0.001		159	102	137	152	92	132	-7	-2.8	<0.001
Saturated fatty acid (g)	18.2	8.9	17.0	17.9	8.9	16.5	-0.2	-1.4	0.02		17.8	8.6	15.3	18.0	8.8	15.4	0.1	0.5	0.01
Monounsaturated fatty acid (g)	23.8	10.7	23.2	21.0	9.3	20.5	-2.8	-11.5	<0.001		22.6	10.1	21.2	20.4	9.2	19.0	-2.2	-9.8	<0.001
Polyunsaturated fatty acid (g)	14.7	6.7	13.3	14.6	6.1	14.1	-0.1	0.7	0.52		12.8	5.1	12.2	13.8	5.8	13.1	0.9	6.8	<0.001
Cholesterol (mg)	334	155	320	327	150	314	-8	-1.8	<0.001		323	184	295	316	173	288	-7	-1.2	<0.001
Water-soluble fiber (g)	2.3	1.5	2.0	3.4	1.8	3.0	1.0	56.8	<0.001		2.3	1.5	2.0	3.2	1.7	2.8	0.9	49.8	<0.001
Water-insoluble fiber (g)	10.3	5.4	9.5	10.6	5.7	9.9	0.3	3.2	0.01		9.6	4.7	8.9	9.8	4.9	8.8	0.2	1.6	0.06
Total dietary fiber (g)	14.6	7.5	13.6	14.4	7.6	13.1	-0.2	-0.9	0.39		13.9	6.8	12.8	13.5	6.9	12.0	-0.4	-3.1	<0.001
NaCl deducted from sodium content (g)	14.3	6.6	13.6	13.4	6.0	13.4	-0.9	-4.9	<0.001		12.6	6.4	11.0	11.5	5.5	10.0	-1.0	-6.9	<0.001

										Female									
	n=113										n=176								
Energy (kcal)	1992	850	1834	1968	788	1837	-24	-0.6	0.01		1803	645	1680	1841	654	1721	38	2.1	<0.001
Protein (g)	80.9	46.0	71.3	75.7	39.3	67.3	-5.2	-5.3	<0.001		71.2	31.3	65.9	70.4	31.2	65.4	-0.8	-1.2	<0.001
Total fat (g)	62.8	36.7	54.5	60.5	32.9	53.1	-2.3	-2.8	<0.001		57.8	29.4	52.5	58.4	29.6	52.1	0.5	0.6	<0.001
Carbohydrate (g)	274	97	259	274	97	263	0	0.3	0.83		249	77	235	254	78	241	5	2.0	<0.001
Sodium (mg)	5308	3111	4718	4985	2866	4434	-323	-4.7	<0.001		4576	2461	4145	4177	2195	3789	-398	-8.1	<0.001
Potassium (mg)	3282	1876	2803	3199	1832	2756	-83	-2.5	<0.001		3072	1489	2739	3024	1468	2712	-48	-1.6	<0.001
Calcium (mg)	682	409	587	624	331	543	-58	-6.9	<0.001		640	374	560	628	379	550	-12	-2.4	<0.001
Phosphorus (mg)	1295	651	1159	1200	585	1079	-95	-7.1	<0.001		1166	482	1048	1133	498	1030	-33	-3.6	<0.001
Iron (mg)	11.8	7.1	10.6	10.0	5.0	9.0	-1.8	-12.5	<0.001		10.2	4.8	9.2	9.2	3.9	8.3	-1.0	-8.6	<0.001
Retinol (*μ*g)	592	697	418	621	701	438	30	11.1	<0.001		490	534	335	525	540	374	35	12.5	<0.001
Alpha-carotene (*μ*g)	579	495	433	768	647	558	188	31.1	<0.001		566	559	412	769	794	562	203	34.2	<0.001
Beta-carotene (*μ*g)	3290	2493	2789	4920	3607	4147	1630	51.4	<0.001		3069	2690	2434	4659	3927	3724	1590	55.0	<0.001
Vitamin B_1_ (mg)	1.21	0.63	1.05	1.14	0.66	0.96	-0.07	-7.6	<0.001		1.20	0.61	1.05	1.10	0.57	0.94	-0.11	-9.5	<0.001
Vitamin B_2_ (mg)	1.68	0.86	1.51	1.59	0.80	1.44	-0.09	-5.2	<0.001		1.63	0.78	1.47	1.55	0.73	1.39	-0.08	-4.7	<0.001
Niacin (mg)	18.0	10.9	15.6	19.8	11.7	17.1	1.8	10.7	<0.001		16.5	8.5	14.9	18.1	9.1	16.3	1.6	9.9	<0.001
Vitamin B_6_ (mg)	1.75	1.05	1.51	1.62	1.01	1.41	-0.13	-7.6	<0.001		1.62	0.81	1.46	1.51	0.73	1.35	-0.12	-6.8	<0.001
Vitamin B_12_ (*μ*g)	11.2	10.9	8.8	10.3	9.3	7.8	-0.9	-6.0	<0.001		9.6	6.2	8.4	8.6	5.3	7.3	-1.0	-7.9	<0.001
Folate (*μ*g)	327	187	277	476	287	419	149	45.7	<0.001		306	166	273	454	237	397	148	49.1	<0.001
Vitamin C (mg)	192	159	156	181	144	150	-11	-4.9	<0.001		189	121	161	181	112	154	-8	-2.9	<0.001
Saturated fatty acid (g)	17.8	9.1	15.4	17.6	8.9	15.5	-0.3	-1.4	0.01		17.5	9.8	15.7	17.7	9.9	15.7	0.2	0.9	<0.001
Monounsaturated fatty acid (g)	23.4	14.0	19.9	20.5	11.7	17.5	-2.9	-11.9	<0.001		21.8	11.9	19.4	19.6	10.7	17.2	-2.2	-10.2	<0.001
Polyunsaturated fatty acid (g)	14.8	10.4	12.4	14.7	9.0	12.6	-0.2	1.2	0.45		12.6	5.9	11.4	13.6	6.6	12.3	1.0	7.0	<0.001
Cholesterol (mg)	316	168	306	309	162	301	-7	-1.5	<0.001		290	171	269	285	164	264	-5	-0.9	<0.001
Water-soluble fiber (g)	2.7	2.0	2.2	3.8	2.5	3.3	1.1	47.5	<0.001		2.7	1.6	2.2	3.6	2.0	3.2	0.9	38.8	<0.001
Water-insoluble fiber (g)	11.3	7.4	9.5	11.7	7.8	10.2	0.4	3.7	<0.001		10.5	5.5	9.3	10.8	5.9	9.5	0.2	1.4	0.01
Total dietary fiber (g)	16.1	10.3	14.1	16.1	10.5	14.2	0.1	0.2	0.73		15.2	7.8	13.7	14.9	8.0	13.0	-0.3	-2.5	0.01
NaCl deducted from sodium content (g)	13.5	7.9	12.0	12.6	7.3	11.2	-0.9	-5.3	<0.001		11.6	6.2	10.5	10.5	5.5	9.5	-1.1	-9.3	<0.001

*: The Fourth Revised Edition of the Standard Tables of Food Composition in Japan

† : The Fifth Revised Edition of the Standard Tables of Food Composition in Japan

‡ : Invidiual intake level calculated with the previous database was subtracted from the intake level calculated with the revised database.

§ : Difference between intake calculated with the previous and revised databases was divided by intake calculated with the previous database at the individual level.

∥ : Statistical difference tested by Student’s t-test

In contrast, revision of the database did not have a substantial effect on the validity of intake levels by FFQ compared to those by DR (Table 2). A greater than 0.1 decline in point estimates of Spearman’s CCs was seen only for the crude intake of vitamin B_1_ and water-soluble fiber in the Cohort I males; in energy-adjusted intake of vitamin B_2_ in Cohort I females; and in crude intake of sodium in Cohort II females. On the other hand, a greater than 0.1 increased point estimate of Spearman’s CCs was observed for the crude intake of crude retinol and polyunsaturated fatty acid in Cohort II females. Confidence intervals of CCs between the previous and revised database overlapped for all nutrients.

**Table 2.** Comparison of Spearman rank correlation coefficients between nutrient intake assessed with dietary records and food frequency questionnaires calculated using 2 databases.

	Cohort I				Cohort II			
	
	Crude		Energy-adjusted		Crude		Energy-adjusted	
			
	Previous*	Revised^†^	Previous*	Revised^†^	Previous*	Revised^†^	Previous*	Revised^†^
	Male							
	n=102				n=174			
Energy	0.55	0.53	-	-	0.34	0.36	-	-
Protein	0.50	0.45	0.30	0.30	0.29	0.28	0.30	0.31
Total fat	0.31	0.34	0.52	0.55	0.26	0.26	0.57	0.57
Carbohydrate	0.71	0.72	0.56	0.66	0.40	0.47	0.59	0.69
Sodium	0.59	0.53	0.41	0.47	0.29	0.25	0.42	0.32
Potassium	0.52	0.51	0.39	0.49	0.33	0.32	0.49	0.48
Calcium	0.65	0.60	0.43	0.54	0.53	0.56	0.65	0.68
Phosphorus	0.61	0.55	0.37	0.45	0.39	0.37	0.49	0.46
Iron	0.52	0.53	0.49	0.44	0.27	0.33	0.54	0.54
Retinol	0.40	0.40	0.22	0.37	0.37	0.37	0.35	0.43
Alpha-carotene	0.47	0.45	0.47	0.51	0.47	0.44	0.50	0.47
Beta-carotene	0.40	0.37	0.41	0.40	0.40	0.39	0.45	0.46
Vitamin B_1_	0.49	0.38	0.40	0.33	0.22	0.28	0.28	0.34
Vitamin B_2_	0.54	0.52	0.34	0.41	0.41	0.42	0.55	0.57
Niacin	0.42	0.36	0.35	0.33	0.34	0.37	0.33	0.35
Vitamin C	0.44	0.47	0.42	0.43	0.38	0.39	0.46	0.48
Saturated fatty acid	0.43	0.47	0.61	0.59	0.42	0.40	0.62	0.62
Monounsaturated fatty acid	0.30	0.33	0.50	0.53	0.26	0.23	0.55	0.53
Polyunsaturated fatty acid	0.16	0.18	0.27	0.39	0.17	0.17	0.44	0.47
Cholesterol	0.42	0.42	0.33	0.33	0.44	0.44	0.47	0.50
Water-soluble fiber	0.48	0.34	0.44	0.38	0.44	0.40	0.54	0.55
Water-insoluble fiber	0.51	0.46	0.43	0.43	0.39	0.40	0.56	0.56
Total dietary fiber	0.50	0.42	0.43	0.41	0.41	0.42	0.57	0.57

	Female							
	n=113				n=176			
Energy	0.44	0.41	-	-	0.22	0.24	-	-
Protein	0.41	0.37	0.27	0.24	0.35	0.34	0.31	0.33
Total fat	0.22	0.20	0.46	0.39	0.31	0.31	0.40	0.46
Carbohydrate	0.56	0.56	0.37	0.45	0.24	0.30	0.39	0.47
Sodium	0.55	0.50	0.48	0.50	0.39	0.32	0.45	0.31
Potassium	0.40	0.35	0.31	0.40	0.40	0.40	0.49	0.50
Calcium	0.53	0.46	0.47	0.45	0.50	0.53	0.64	0.68
Phosphorus	0.49	0.44	0.42	0.44	0.41	0.41	0.54	0.55
Iron	0.41	0.38	0.33	0.38	0.39	0.44	0.51	0.55
Retinol	0.35	0.32	0.43	0.39	0.44	0.42	0.47	0.49
Alpha-carotene	0.46	0.42	0.50	0.48	0.52	0.51	0.52	0.53
Beta-carotene	0.30	0.30	0.32	0.33	0.47	0.48	0.47	0.48
Vitamin B_1_	0.31	0.29	0.41	0.32	0.33	0.31	0.32	0.35
Vitamin B_2_	0.43	0.35	0.45	0.31	0.46	0.49	0.55	0.58
Niacin	0.27	0.24	0.15	0.11	0.22	0.18	0.22	0.21
Vitamin C	0.31	0.33	0.22	0.30	0.42	0.46	0.44	0.47
Saturated fatty acid	0.26	0.33	0.60	0.55	0.42	0.41	0.51	0.54
Monounsaturated fatty acid	0.13	0.14	0.44	0.36	0.31	0.30	0.37	0.44
Polyunsaturated fatty acid	0.16	0.11	0.24	0.22	0.23	0.22	0.33	0.37
Cholesterol	0.31	0.29	0.35	0.32	0.49	0.46	0.47	0.49
Water-soluble fiber	0.40	0.30	0.36	0.32	0.42	0.45	0.46	0.52
Water-insoluble fiber	0.45	0.39	0.40	0.44	0.44	0.46	0.50	0.54
Total dietary fiber	0.44	0.35	0.40	0.41	0.42	0.46	0.49	0.53

* : The Fourth Revised Edition of the Standard Tables of Food Composition in Japan

† : The Fifth Revised Edition of the Standard Tables of Food Composition in Japan

Likewise, the validity of the FFQ was not influenced by the database revision when compared to biomarker data (Table 3). For those nutrients for which biomarkers are a good indicator of dietary intake, such as serum polyunsaturated fatty acid, carotenoids, and urinary sodium and potassium, CCs for the estimated intake calculated by the previous and revised databases were similar. As with comparison by DR, confidence intervals of CCs between the previous and revised database overlapped for all nutrients. Moreover, reproducibility (FFQV vs. FFQR) was also not altered by the database revision (data not shown).

**Table 3.** Spearman rank correlation coefficients between nutrient intakes assessed with a food frequency questionnaire and corresponding biomarker

Biomarker		Male							Female					
	
	n	Crude		Adjusted^§^		Adjusted^∥^		n	Crude		Adjusted^§^		Adjusted^∥^	
					
		Previous*	Revised^†^	Previous*	Revised^†^	Previous*	Revised^†^		Previous*	Revised^†^	Previous*	Revised^†^	Previous*	Revised^†^
Serum phospholipid														
Saturated fatty acid	88	-0.20	-0.17	-0.13	-0.14	-0.01	-0.02	49	0.02	-0.01	-0.05	-0.07	-0.20	-0.14
Monounsaturated fatty acid	88	-0.16	-0.13	0.05	0.08	0.19	0.19	49	-0.11	-0.11	-0.42	-0.41	-0.25	-0.07
Polyunsaturated fatty acid	88	0.31	0.27	0.16	0.12	0.13	0.09	49	-0.21	-0.13	-0.12	0.09	-0.40	-0.34

Serum														
Alpha-carotene	86	0.37	0.38	0.38	0.40	-	-	99	0.30	0.28	0.32	0.29	-	-
Beta-carotene	86	0.28	0.28	0.27	0.25	-	-	99	0.12	0.11	0.07	0.10	-	-
Cryptxanthin	86	0.48	0.50	0.48	0.52	-	-	99	0.40	0.35	0.36	0.36	-	-

Plasma														
Folate	87	0.05	-0.05	0.26	0.19	-	-	-	-	-	-	-	-	-
Vitamin B_6_	87	0.17	0.10	0.23	0.19	-	-	-	-	-	-	-	-	-
Vitamin B_12_	87	0.001	-0.01	0.06	0.05	-	-	-	-	-	-	-	-	-
Vitamin C	88	-0.004	0.03	-0.11	-0.08	-	-	100	0.05	0.07	-0.07	-0.03	-	-

Urine														
Sodium	33	0.21	0.28	0.11	0.22	0.40	0.42	61	0.31	0.30	0.38	0.36	0.37	0.30
Potassium	33	0.23	0.27	0.23	0.20	0.40	0.40	61	0.18	0.16	0.11	0.09	0.25	0.24

* : The Forth Revised Edition of the Standard Tables of Food Composition in Japan

† : The Fifth Revised Edition of the Standard Tables of Food Composition in Japan

§ : Energy was adjusted by residual method.

∥ : For each fatty acid intake, percentage of total fatty acid intake was used. For sodium and potassium, urinary excretion values were adjusted for creatine, and intake values were adjusted for energy by the residual method.

Estimated intake according to DRs and FFQ, as well as Spearman’s CC, for nutrients which were newly included in the revised database and never previously evaluated for validity and reproducibility are presented in Table 4. Spearman’s CC for the estimation of most of these nutrients by FFQ indicated moderate validity (Spearman’s CC=0.3-0.6), except for vitamins D and E, which indicated slightly lower validity.

**Table 4.** Intake of new nutrients in the revised nutrient database according to DRs for 28 or 14 days and FFQ, and Spearman rank correlation coefficients (CC) between DR and FFQv, and FFQr and FFQv.

	Cohort I											Cohort II									
	
	DR			FFQv			Spearman CC		Spearman CC			DR			FFQv			Spearman CC		Spearman CC	
							DR and FFQv		FFQr and FFQv									DR and FFQv		FFQr and FFQv	
							
	Mean	SD	Median	Mean	SD	Median	Crude	Energy- adjusted	Crude	Energy- adjusted		Mean	SD	Median	Mean	SD	Median	Crude	Energy- adjusted	Crude	Energy- adjusted
											Male										
	n=102											n=174									
Magnesium (mg)	349	67	348	323	124	301	0.51	0.46	0.59	0.62		349	66	345	294	109	271	0.32	0.45	0.68	0.70
Zinc (mg)	10.7	2.1	10.6	9.7	3.2	9.4	0.58	0.50	0.59	0.45		10.2	1.7	10.4	9.0	3.0	8.5	0.33	0.44	0.62	0.67
Copper (mg)	1.53	0.36	1.52	1.47	0.54	1.42	0.66	0.64	0.71	0.62		1.48	0.28	1.48	1.31	0.44	1.24	0.39	0.60	0.69	0.70
Manganese (mg)	4.28	1.02	4.34	5.14	2.09	4.63	0.60	0.45	0.71	0.69		5.56	3.43	4.83	4.54	1.65	4.20	0.35	0.40	0.72	0.66
Retinol equivalents (*μ*g)	1193	600	1034	1541	988	1401	0.42	0.47	0.54	0.83		1113	478	1016	1398	793	1222	0.36	0.43	0.65	0.53
Cryptoxanthin (*μ*g)	364	318	280	1160	1159	870	0.48	0.43	0.57	0.49		454	359	348	1373	1655	846	0.48	0.48	0.55	0.52
Beta-carotene equivalents (*μ*g)	4456	1666	4007	5383	4213	4786	0.38	0.43	0.51	0.44		4634	1803	4331	5118	3979	4144	0.41	0.47	0.61	0.56
Vitamin D (*μ*g)	13.6	5.3	13.3	13.0	10.1	9.9	0.39	0.26	0.62	0.77		12.7	5.6	11.7	10.8	7.7	8.7	0.30	0.32	0.62	0.56
Alpha-tocopherol (mg)	8.8	1.7	8.9	8.1	3.9	7.6	0.28	0.37	0.57	0.65		8.5	1.8	8.4	7.7	3.7	7.1	0.18	0.24	0.61	0.58
Beta-tocopherol (mg)	0.4	0.1	0.4	0.4	0.2	0.4	0.09	0.35	0.49	0.60		0.4	0.1	0.4	0.4	0.2	0.3	0.12	0.25	0.60	0.62
Gamma-tocopherol (mg)	12.8	3.1	12.4	12.8	5.6	12.0	0.16	0.33	0.57	0.62		12.4	3.1	12.1	11.9	5.2	11.4	0.10	0.20	0.64	0.58
Delta-tocopherol (mg)	3.6	0.9	3.5	3.5	1.7	3.3	0.25	0.44	0.64	0.67		3.3	0.9	3.2	3.0	1.4	2.9	0.30	0.42	0.66	0.63
Vitamin K (*μ*g)	276.0	98.0	261.8	342.8	244.6	260.9	0.49	0.53	0.69	0.87		263.1	95.2	251.9	290.7	226.7	228.8	0.50	0.57	0.69	0.66
Vitamin B_6_ (mg)	1.8	0.4	1.8	1.8	0.8	1.7	0.47	0.45	0.60	0.56		1.8	0.4	1.8	1.7	0.7	1.6	0.36	0.36	0.69	0.59
Vitamin B_12_ (*μ*g)	12.2	5.1	12.1	11.0	7.5	8.8	0.48	0.33	0.64	0.71		11.0	3.8	10.6	9.5	5.5	8.1	0.35	0.35	0.66	0.58
Folate (*μ*g)	425	103	427	473	231	444	0.49	0.40	0.65	0.77		467	156	443	421	190	370	0.33	0.50	0.67	0.62
Pantothenic acid (mg)	7.42	1.55	7.51	7.73	3.13	7.15	0.61	0.69	0.61	0.60		7.25	1.31	7.17	7.24	3.04	6.66	0.39	0.54	0.64	0.71
NaCl deducted from sodium content (g)	12.6	3.0	12.4	13.4	6.0	13.4	0.53	0.47	0.60	0.72		11.1	2.7	10.7	11.5	5.5	10.0	0.24	0.30	0.57	0.55

											Female										
	n=113											n=176									
Magnesium (mg)	295	63	296	306	154	281	0.39	0.42	0.71	0.61		300	51	297	285	121	256	0.32	0.45	0.65	0.61
Zinc (mg)	8.7	1.6	8.8	8.8	3.7	8.1	0.44	0.35	0.70	0.43		8.4	1.3	8.5	8.2	3.2	7.6	0.32	0.40	0.62	0.59
Copper (mg)	1.27	0.28	1.27	1.40	0.69	1.27	0.46	0.58	0.77	0.74		1.26	0.21	1.26	1.28	0.49	1.16	0.40	0.58	0.65	0.62
Manganese (mg)	3.49	0.83	3.38	4.45	1.95	3.99	0.33	0.42	0.68	0.60		4.72	2.27	4.26	4.46	1.77	4.10	0.40	0.39	0.68	0.62
Retinol equivalents (*μ*g)	1127	548	965	1625	1163	1321	0.25	0.31	0.50	0.43		1058	393	992	1507	998	1301	0.39	0.44	0.60	0.52
Cryptoxanthin (*μ*g)	496	391	455	1454	1474	1036	0.35	0.29	0.58	0.58		597	329	570	1728	1574	1271	0.31	0.31	0.50	0.44
Beta-carotene equivalents (*μ*g)	4470	1730	4042	6020	4372	4863	0.30	0.31	0.47	0.44		4553	1545	4315	5892	4541	4881	0.44	0.44	0.66	0.55
Vitamin D (*μ*g)	11.5	4.3	11.6	12.7	13.7	9.3	0.42	0.38	0.62	0.43		9.8	3.5	9.7	10.0	6.9	8.1	0.29	0.28	0.62	0.52
Alpha-tocopherol (mg)	8.2	1.7	7.9	8.6	5.6	7.2	0.29	0.50	0.60	0.49		7.7	1.4	7.6	8.0	4.2	7.1	0.25	0.37	0.59	0.35
Beta-tocopherol (mg)	0.4	0.1	0.4	0.4	0.2	0.3	0.20	0.40	0.70	0.61		0.4	0.1	0.3	0.4	0.2	0.3	0.15	0.27	0.62	0.55
Gamma-tocopherol (mg)	11.6	2.6	11.3	13.3	8.2	11.6	0.20	0.42	0.73	0.62		11.1	2.6	11.0	12.2	6.1	11.1	0.24	0.43	0.64	0.50
Delta-tocopherol (mg)	3.2	0.8	3.2	3.6	2.3	3.3	0.36	0.47	0.77	0.68		3.0	0.8	2.9	3.2	1.6	2.9	0.36	0.49	0.62	0.46
Vitamin K (*μ*g)	256.5	91.2	245.2	368.2	260.9	313.7	0.38	0.43	0.75	0.69		255.6	87.5	245.6	332.5	249.8	253.0	0.52	0.57	0.68	0.63
Vitamin B_6_ (mg)	1.4	0.3	1.5	1.6	1.0	1.4	0.43	0.47	0.68	0.59		1.4	0.2	1.4	1.5	0.7	1.4	0.34	0.40	0.66	0.59
Vitamin B_12_ (*μ*g)	9.9	3.8	10.0	10.3	9.3	7.8	0.39	0.34	0.66	0.49		8.3	2.5	8.3	8.6	5.3	7.3	0.30	0.27	0.58	0.49
Folate (*μ*g)	389	106	380	476	287	419	0.29	0.35	0.72	0.63		426	112	417	454	237	397	0.42	0.48	0.64	0.58
Pantothenic acid (mg)	6.43	1.30	6.60	7.47	3.83	7.00	0.45	0.43	0.69	0.57		6.36	1.06	6.33	7.09	3.16	6.38	0.46	0.61	0.57	0.62
NaCl deducted from sodium content (g)	11.0	2.6	10.6	12.6	7.3	11.2	0.50	0.50	0.75	0.76		9.5	2.2	9.5	10.5	5.5	9.5	0.32	0.31	0.66	0.66

DR: 28-day dietary records

FFQv: food frequency questionnaire for validity

FFQr: food frequency questionnaire for reproducibility

SD: standard deviation

## DISCUSSION

We evaluated the impact of revision of the food composition database on the estimation of energy and nutrient intake by the FFQ in the JPHC Study, and its validity. The results of recalculation using the revised food composition table showed that, notwithstanding a significant impact on the estimation of individual intake levels for some nutrients, the revision had little substantial influence on the validity of individual rankings by estimated nutrient intake.

We observed major decreases in the intake of iron, vitamin B_1_, and monounsaturated fatty acid, and increases in that of carotene, retinol, niacin and water-soluble fiber as a result of revision of the food composition database. These results are in agreement with several previous studies which investigated changes in nutrient intake estimates in Japan,^5^^,^^6^ with the exception of the estimated intake of fatty acids and cholesterol, which showed a drastic decrease in one study but no decrease or radical change in the present study. This difference is likely due to our supplementation of missing values in the food composition database. Influence on nutrient intake did not differ among age groups because, unlike the previous study, the age range of our subjects did not include subjects aged below 45 years.^6^ In addition, changes in intake by database revision were also computed for nutrient intake according to the DRs (data not shown). The percentages of differences between nutrient intake calculated using the two databases according to the DR were closely similar to those assessed by the FFQ; in other words, the degree of over- or underestimation of intake by the FFQ was not modified by revision of the database.

Validity levels of the FFQ were moderate to high for the estimation of energy and of most nutrient intakes. These levels were not changed by revision of the food composition table in subjects of either Cohort I or II. Similar results between the two cohorts suggested the possibility that the results could be generalized; that is, revision of the nutrient database might not have affected the validity of the FFQ as assessed in an external population.

In general, DRs provide the best available comparison method,^1^ and are often used as the gold standard in validation studies of FFQs. However, nutrient intake calculated using an FFQ is not completely independent from that using a DR because the same food composition table is used to calculate nutrient intakes for both methods. The present results therefore appear unsurprising, given that the reference method was also calculated using the revised food composition tables. To compensate for this limitation, validity was also tested using biomarkers as references, which are totally independent of dietary assessments. Results for these also indicated that validity was only little influenced by revision of the nutrient database. Validity of the estimation of fatty acids, B group vitamins, and vitamin C was markedly low when biomarkers were used as references with either database, however, because biomarkers are not good indicators of the long-term habitual intake of these nutrients.^15^^,^^16^ The second limitation of this study was that we did not conduct equivalence testing for two correlation coefficients. Although this is required to show equivalence, it is generally not done because of the complexity of estimating variance components and constituting a confidence interval from the statistics for the ratio and difference between two correlation coefficients. Comparison using the point estimates and confidence intervals of each correlation coefficient revealed relatively low differences for each, and on this albeit informal basis we evaluated the two correlation coefficients as being similar.

In conclusion, the validity of the FFQ used in the JPHC Study to estimate nutrient intake was not influenced by revision of the Standard Tables of Food Composition in Japan. Associations between disease and nutrients would therefore be consistent between the databases as long as nutrient intake was used for ranking.

## APPENDIX

The investigators and their affiliations in the validation study of the self-administered food frequency questionnaire in the JPHC Study (the JPHC FFQ Validation Study Group) at the time of the study were: Tsugane S, Sasaki S, and Kobayashi M, Epidemiology and Biostatistics Division, National Cancer Center Research Institute East, Kashiwa; Sobue T, Yamamoto S, and Ishihara J, Cancer Information and Epidemiology Division, National Cancer Center Research Institute, Tokyo; Akabane M, Iitoi Y, Iwase Y, and Takahashi T, Tokyo University of Agriculture, Tokyo; Hasegawa K, and Kawabata T, Kagawa Nutrition University, Sakado; Tsubono Y, Tohoku University, Sendai; Iso H, Tsukuba University, Tsukuba; Karita S, Teikyo University, Tokyo; the late Yamaguchi M, and Matsumura Y, National Institute of Health and Nutrition, Tokyo.
